# Complete Genome Sequence of Teviot Paramyxovirus, a Novel Rubulavirus Isolated from Fruit Bats in Australia

**DOI:** 10.1128/genomeA.00177-15

**Published:** 2015-04-16

**Authors:** Amy L. Burroughs, Mary Tachedjian, Gary Crameri, Peter A. Durr, Glenn A. Marsh, Lin-Fa Wang

**Affiliations:** a Australian Animal Health Laboratory, Commonwealth Scientific and Industrial Research Organization, East Geelong, Victoria, Australia; b School of Veterinary Science, University of Queensland, Gatton, Queensland, Australia; c Duke-NUS Graduate Medical School, Singapore

## Abstract

The causative agents of a number of emerging zoonotic diseases have been identified as paramyxoviruses originating in bats. We report here the complete genome sequence of two Teviot paramyxoviruses, novel rubulaviruses isolated from urine samples collected from pteropid bats in Australia. The zoonotic potential of Teviot paramyxovirus is undetermined.

## GENOME ANNOUNCEMENT

Bats (order *Chiroptera*) are reservoirs of many emerging zoonotic viruses (1). Pertinent examples include Hendra and Nipah viruses, members of the family *Paramyxoviridae*, genus *Henipavirus* (2). Of the known paramyxoviruses of genus *Rubulavirus*, a significant proportion has been isolated and/or detected from bats (3–12), particularly fruit bats. Evidence suggests that bat rubulaviruses are capable of crossing species barriers (6, 13, 14) and causing disease in humans (14).

As part of a surveillance study, Teviot paramyxovirus (TevPV) was isolated from pooled urine samples collected from *Pteropus alecto* fruit bats at Cedar Grove, Queensland, Australia, in September 2009 (3). The complete genome sequence of the Cedar Grove TevPV isolate was obtained using the Roche 454-GS FLX system (454 Life Sciences, Branford, CT, USA) following procedures previously reported by our group (15). In November 2011, a second isolate of TevPV was obtained, also from pooled urine samples collected from *P. poliocephalus* fruit bats in Geelong, Victoria, which is over 1,800 km away from Cedar Grove. The complete genome sequence of this isolate was obtained with the Illumina MiSeq platform following protocols from the supplier. A combination of *de novo* assembly and read mapping of raw reads to a Tioman virus template was used to obtain the TevPV consensus sequence. Sequences at the 5′ (6 nucleotides [nt]) and 3′ (2 nt) ends were inferred from the Tioman virus genome due to their close genetic relationship. Open reading frames were also extrapolated from those of Tioman virus.

The genome of TevPV is 15,522 nt in length and is a multiple of six, conforming to the rule of six for viruses of the subfamily *Paramyxovirinae* (16, 17). Phylogenetic analysis of the complete genome sequence confirmed that it is most closely related to Tioman virus, which had previously been inferred from partial sequence of the L gene (12). Genome organization was typical of rubulaviruses, with six genes (3′-NP-P/V-M-F-HN-L-5′) encoding at least seven different proteins. Genes were bound by conserved transcriptional start and stop signals and separated by variable intergenic regions. The Geelong isolate showed remarkable sequence identity to the isolate obtained from Queensland in 2009, with only 0.65% nucleotide variation across the entire genome. The P/V gene of rubulaviruses undergoes RNA editing, and the Illumina reads enabled this to be assessed. The RNA editing site was identified between nucleotide positions 2474 and 2475 of the positive sense genome TTTAAGA*GGGGGGATT. In most cases, there were either 0-G or 2-G nontemplated insertions in the P gene mRNA coding for the V and P proteins, respectively. No reads contained a single G insertion, which for Tioman virus codes for the W protein.

The zoonotic potential of TevPV is undetermined. The availability of complete genome sequences of two isolates of a novel bat rubulavirus, TevPV, improves our understanding of the diversity of viruses circulating in bat populations and provides a target in zoonotic disease surveillance.

### Nucleotide sequence accession numbers.

The complete genomes of the Cedar Grove and Geelong isolates of TevPV have been deposited in GenBank with the accession numbers KP271124 and KP271123, respectively.
